# Comparative Analyses of Antiviral Potencies of Second-Generation Integrase Strand Transfer Inhibitors (INSTIs) and the Developmental Compound 4d Against a Panel of Integrase Quadruple Mutants

**DOI:** 10.3390/v17010121

**Published:** 2025-01-16

**Authors:** Steven J. Smith, Xue Zhi Zhao, Stephen H. Hughes, Terrence R. Burke

**Affiliations:** 1 Chemical Biology Laboratory, Center for Cancer Research, National Cancer Institute, National Institutes of Health, Frederick, MD 21702, USA; xuezhi.zhao@nih.gov (X.Z.Z.); burkete@nih.gov (T.R.B.J.); 2 HIV Dynamics and Replication Program, Center for Cancer Research, National Cancer Institute, National Institutes of Health, Frederick, MD 21702, USA; hughesst@mail.nih.gov

**Keywords:** integrase, resistance, potency, mutations, mutants

## Abstract

Second-generation integrase strand transfer inhibitors (INSTIs) are strongly recommended for people living with HIV-1 (PLWH). The emergence of resistance to second-generation INSTIs has been infrequent and has not yet been a major issue in high-income countries. However, the delayed rollouts of these INSTIs in low- to middle-income countries during the COVID-19 pandemic combined with increased transmission of drug-resistant mutants worldwide are leading to an increase in INSTI resistance. Herein, we evaluated the antiviral potencies of our lead developmental INSTI 4d and the second-generation INSTIs dolutegravir (DTG), bictegravir (BIC), and cabotegravir (CAB) against a panel of IN quadruple mutants. The mutations are centered around G140S/Q148H, including positions L74, E92, and T97 combined with E138A/K/G140S/Q148H. All of the tested INSTIs lose potency against these IN quadruple mutants compared with the wild-type IN. In single-round infection assays, compound 4d retained higher antiviral potencies (EC_50_ values) than second-generation INSTIs against a subset of quadruple mutants. These findings may advance understanding of mechanisms that contribute to resistance and, in so doing, facilitate development of new INSTIs with improved antiviral profiles.

## 1. Introduction

**Figure 1:**
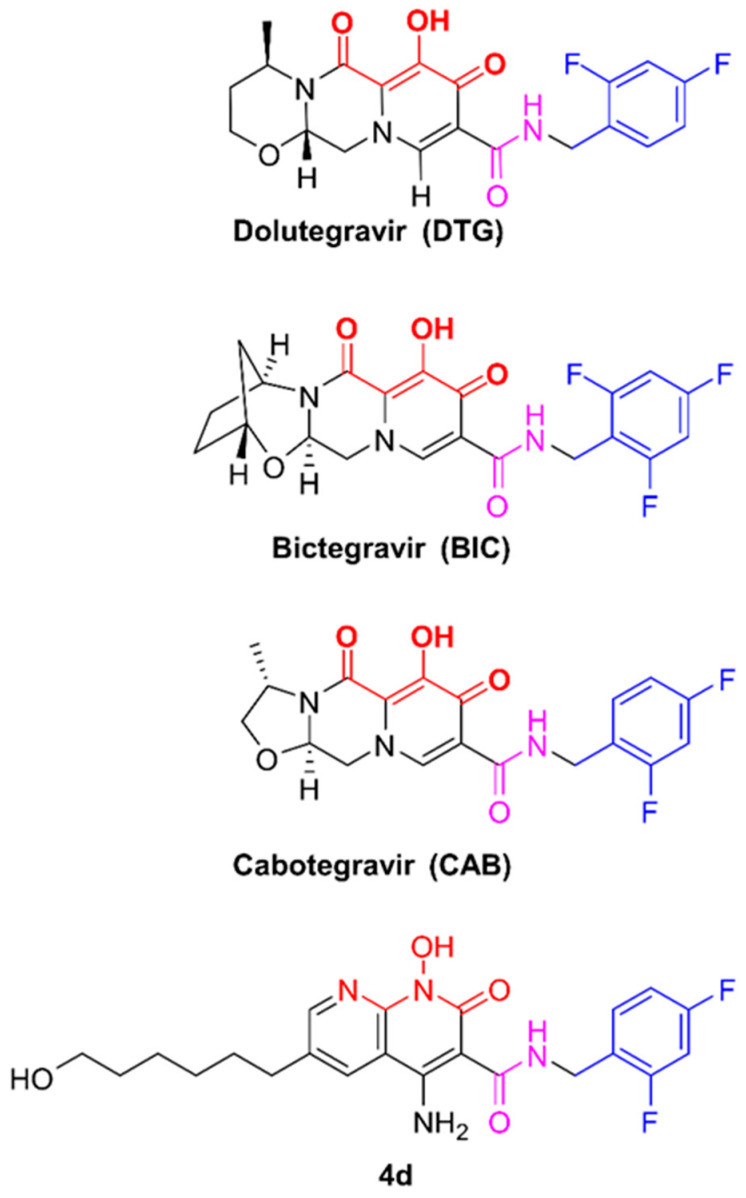
Chemical structures of INSTIs used in this study. Chemical structures of FDA-approved second-generation INSTIs and compound 4d are depicted. Atoms shown in red represent the chelating motifs, while the linker group is in purple and the halogenated benzyl moiety in blue.

Integrase (IN) strand transfer inhibitors (INSTIs) have emerged as leading therapeutics to treat HIV-1 infections and reduce HIV-1 transmission when used in pre-exposure prophylaxis (PrEP). The FDA-approved second-generation INSTIs dolutegravir (DTG), bictegravir (BIC), and cabotegravir (CAB) (Figure 1) are usually prescribed in combination with additional classes of antiretroviral drugs (cARTs). These second-generation INSTIs feature a tricyclic scaffold with a “di-keto” motif. A halogenated benzyl moiety is attached to the scaffold via an optimized linker group. Although the first-generation INSTIs possess the di-keto motif, this is on either a mono- or bicyclic scaffold with chemical modifications that protrude from these scaffolds and interact favorably with wild-type (WT) HIV-1 IN. Structural studies have revealed the importance of a tricyclic scaffold and a linker group of the second-generation INSTIs, as it can bind more favorably to a mutated IN active site [1].

The development of resistance to second-generation INSTIs has been relatively infrequent, particularly if these drugs are used in combination therapies in people living with HIV-1 (PLWH) who are drug naïve [2]. Although resistance pathways to second-generation INSTIs are still being defined, it appears that resistance can develop along at least four distinct pathways: G118R, G140A/S/Q148H/K/R, N155H, and R263K [1].

The emergence of resistance in PLWH in high-income countries is less concerning when compared to low- to middle-income countries due to the availability of second-generation INSTIs [3,4]. The roll-out of the second-generation INSTIs in low- to middle-income countries has been delayed because of the COVID-19 pandemic and the logistics of distributing oral medicines [5,6]. In addition, CAB, which is being used in long-acting formulations that have shown promising results, has a long pharmacokinetic tail. Prolonged suboptimal concentrations of CAB can allow for HIV-1 replication that may select for resistance [7,8,9]. Moreover, transmission of drug-resistant IN mutants could be a major issue in the future not only for first-generation INSTI-resistant mutants but also for mutants that are resistant to second-generation INSTIs [10,11]. It has been well-documented that PLWH whose virus carries the IN mutations G140S/Q148H or other IN mutants with different combinations of mutations at G140 and Q148 can have high failure rates when prescribed DTG. Additionally, additional IN mutations that cause resistance to DTG can arise when PLWH are switched to a DTG-based salvage therapy [12,13,14].

**Figure 2:**
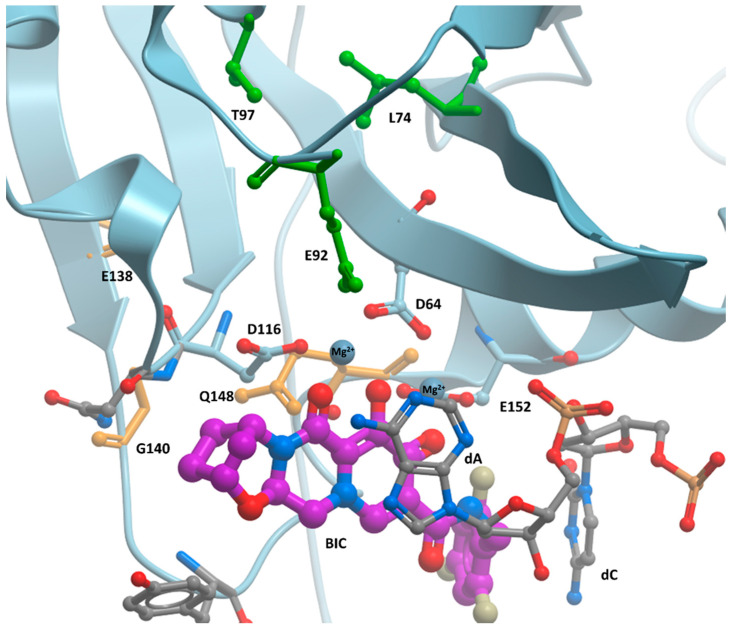
IN residues that undergo mutation during INSTI resistance development. BIC (in purple) in the active site of the HIV-1 intasome (IN is in light blue, while viral DNA end is shown in gray; PDB: 6PUW). Mutations to IN residues depicted in orange are frequently selected by INSTIs and are often found in INSTI-resistant IN double and triple mutants. IN resistance mutations also arise in the residues shown in green. Mutations at these positions can be a part of the IN quadruple mutants that have been analyzed in this study. All residues and nucleotides are labeled in black.

We are currently focused on developing INSTIs that have improved retention of antiviral potencies against mutants in the clinically important G140A/S and Q148H/K/R resistance pathway [15]. Additional mutations, described in previous clinical studies, can arise at IN positions L74, E92, T97, and E138 (Figure 2) [16,17,18,19]. We have extended our previous studies by measuring the EC_50_ values of our lead developmental compound, 4d (Figure 1), and the second-generation INSTIs DTG, BIC, and CAB in single-round infection assays against a panel of eight IN quadruple mutants that have mutations at positions E138A/K and G140S/Q148H and a fourth mutation at IN positions L74, E92, or T97 (Figure 1). Unlike the second-generation INSTIs, 4d features a naphthyridine ring system with protrusions at the 4’ (amino group) and 6’ (hexanol) positions; however, the linker group connecting halogenated benzyl moiety to its scaffold is similar to DTG [20]. All four INSTIs failed to retain antiviral potency against the mutants in this panel. However, 4d and BIC retained greater potency against the mutants than either DTG or CAB, and, in some cases, 4d outperformed BIC, retaining greater potency against some quadruple IN mutants in our assays. In particular, CAB, which is an important component in long-acting formulations, was largely ineffective against this panel of IN quadruple mutants. The ability of 4d to retain considerable antiviral potency against several complex IN mutants is encouraging, and the compound is currently undergoing the initial stages of preclinical testing.

## 2. Materials and Methods

### 2.1. Vector Constructs

To make IN mutants, the vector pNLNgoMIVR-ΔENV.LUC, which has been described previously [16], was used to produce the new mutants analyzed in this study. The IN open reading frame was removed from pNLNgoMIVR-ΔENV.LUC through digestion with KpnI and SalI, and the resulting fragment was inserted between the KpnI and SalI sites of pBluescript KS+. Using this construct as the WT template, the following HIV-1 IN mutants were prepared using the QuikChange II XL site-directed mutagenesis kit (Agilent Technologies, Santa Clara, CA, USA): E92Q/E138A/G140S/Q148H, E92Q/E138K/G140S/Q148H, T97A/E138A/140S/Q148H, T97A/E138K/140S/Q148H, L74M/E138A/G140S/Q148H, L74M/E138K/G140S/Q148H, L74I/E138A/G140S/Q148H, and L74I/E138K/G140S/Q148H. To construct the aforementioned IN quadruple mutants, the IN mutants E138A/G140S/Q148H and E138K/G140S/Q148H, which have been previously constructed, were used as templates in KS modifier constructs [21]. To make the fourth mutation, the proper oligonucleotides for L74I, L74M, E92Q, or T97A were used. The following sense oligonucleotides were used with matching cognate antisense oligonucleotides (Integrated DNA Technologies, Coralville, IA, USA) in the mutagenesis: L74I, 5′-TTAGAAGGAAAAGTTATCATCGTAGCAGTTCATGTAGCC-3′; L74M, 5′-TTAGAAGGAAAAGTTATCATGGTAGCAGTTCATGTAGCC-3′; E92Q, 5′-GCAGAAGTAATTCCAGCACAGACAGGGCAAGAAACAGCA-3′; and T97A, 5′-GCAGAGACAGGGCAAGAAGCAGCATACTTCCTCTTAAAA-3′. After the mutagenesis, the DNA sequence of each construct was determined. The mutated IN coding sequences from pBluescript KS+ were then subcloned into pNLNgoMIVR-ΔEnv.LUC (between the KpnI and SalI sites) to produce mutant HIV-1 constructs, which were also checked through DNA sequencing.

### 2.2. Cell-Based Assays

The human osteosarcoma (HOS) cell line was obtained from Dr. Richard Schwartz (Michigan State University, East Lansing, MI, USA) and grown in Dulbecco’s modified Eagle’s medium (Invitrogen, Carlsbad, CA, USA) supplemented with 5% (*v*/*v*) fetal bovine serum, 5% newborn calf serum, and penicillin (50 units/mL) plus streptomycin (50 µg/mL; Quality Biological, Gaithersburg, MD, USA). As briefly summarized below, single-round infectivity assays were used to determine antiviral activities of the compounds [22].

### 2.3. Pseudotyped HIV-1 Production

Briefly, VSV-g-pseudotyped HIV was produced through transfection of 293 cells, as mentioned earlier [22]. On the day prior to transfection, 293 cells were plated on 100 mm diameter dishes at a density of 1.5 × 10^6^ cells per plate. Then, 293 cells were transfected with 16 µg of pNLNgoMIVR-ΔLUC and 4 µg of pHCMV-g (obtained from Dr. Jane Burns, University of California, San Diego, CA, USA) using the calcium phosphate method. At approximately 6 h after the calcium phosphate precipitate was added, 293 cells were washed twice with phosphate-buffered saline (PBS) and incubated with fresh media for 48 h. The virus-containing supernatants were then harvested, clarified through low-speed centrifugation, filtrated, and diluted for preparation in antiviral infection assays.

### 2.4. Antiviral Assays to Determine EC_50_

On the day prior to screen, HOS cells were seeded in a 96-well luminescence cell culture plate at a density of 4000 cells in 100 µL per well. On the day of the screen, cells were treated with compounds from a concentration range of 5000 nM to 0.1 nM using 11 serial dilutions and then incubated at 37 °C for 3 h. After compound incorporation into the cells, 100 µL of virus stock diluted to achieve a luciferase signal between 0.2 and 1.5 Relative Luciferase Units (RLUs) was added to each well and further incubated at 37 °C for 48 h. Infectivity was measured by using the Steady-lite plus luminescence reporter gene assay system (PerkinElmer, Waltham, MA, USA). Luciferase activity was measured by adding 100 µL of Steady-lite plus buffer (PerkinElmer) to the cells, incubating at room temperature for 20 min, and measuring luminescence using a microplate reader. Antiviral activities were normalized to the infectivity in cells that featured the absence of target compounds. KaleidaGraph (Synergy Software, Reading, PA, USA) was used to perform non-linear regression analysis on the data. EC_50_ values, which are the concentrations of the inhibitors that reduce the activity of the luciferase carried by the HIV vector by 50 percent in a single-round infection, were determined from the fit model [22].

### 2.5. Single-Round Infection Assays

A modified version of the single-round infectivity assay was used to determine the infection capacity of the INSTI-resistant mutant vectors. Briefly, 200 ng of a WT or INSTI-resistant mutant HIV-1 virus was added to each well in 96-well plates and incubated for 48 h, and the luciferase activity was measured as mentioned above. The luciferase activity of the WT virus was set to 100%, and the infectivity of the mutant viruses was measured as a percentage of the WT [23].

## 3. Results

### 3.1. Antiviral Potencies of Compound 4d and the Second-Generation INSTIs Against a Panel of IN Quadruple Mutants with Mutations at IN Positions L74, E92, or T97 Combined with E138A/K/G140S/Q148H

We have previously shown that INSTI 4d retains antiviral potency against simpler mutants, as measured by EC_50_ values in a single-round assay [16,20,23,24]. We employ single-round assays because they are a rapid and reproducible way to measure antiviral potency. Because potency is measured in a single round of infection, the replication capacity of the IN mutant does not affect the result, which is not the case in multi-round assays. Compound 4d and the three FDA-approved drugs were tested against panels of IN mutants [16,20,23,24]. Because of their potential to cause resistance to the approved second-generation INSTIs, we constructed a series of IN quadruple mutants that included the following mutations at IN positions L74I/M, E92Q, or T97A/E138A/K/G140S/Q148H. The following eight IN quadruple mutants were tested in single-round infection assays (Table 1): E92Q/E138A/G140S/Q148H, E92Q/E138K/G140S/Q148H, T97A/E138A/140S/Q148H, T97A/E138K/140S/Q148H, L74M/E138A/G140S/Q148H, L74M/E138K/G140S/Q148H, L74I/E138A/G140S/Q148H, and L74I/E138K/G140S/Q148H. None of the INSTIs we tested retained high antiviral potencies against all of the IN quadruple mutants, which was contradictory (except for CAB) when compared to their antiviral potencies against IN triple mutants E138A/K/G140S/Q148 (see discussion) [16,21]. In fact, none of the INSTIs exhibited antiviral potencies < 20 nM against these IN mutants. CAB was the least effective against this panel of IN mutants (all potencies > 500 nM). We also saw large reductions in potencies of DTG, BIC, and 4d; however, BIC and 4d retained significantly greater potency than DTG and CAB. While BIC and 4d displayed potencies < 100 nM against the majority of IN mutants in the panel, DTG displayed potencies < 100 nM against only two IN mutants.

The IN quadruple mutants E92Q/E138A/G140S/Q148H and E92Q/E138K/G140S/Q148H both caused the largest reduction in susceptibility to the INSTIs. Both BIC and 4d exhibited similar potencies against E92Q/E138A/G140S/Q148H (102.5 ± 13.6 nM and 120.9 ± 22.6 nM, respectively). However, BIC (78.5 ± 10.9 nM) displayed an improved potency against E92Q/E138K/G140S/Q148H when compared to DTG (126.5 ± 31.0 nM; *p*-value < 0.05), 4d (179.2 ± 27.6 nM), and CAB (513.8 ± 77.9 nM). Compound 4d showed improved potency (92.5 ± 4.7 nM) against the quadruple mutant T97A/E138A/140S/Q148H when compared to BIC (126.0 ± 6.0 nM; *p*-values < 0.001), while both 4d and BIC exhibited similar potencies against T97A/E138K/140S/Q148H (52.1 ± 8.8 nM and 64.3 ± 4.5 nM, respectively). Compound 4d was more effective than the other INSTIs in the panel at inhibiting the remaining four IN quadruple mutants. Compound 4d inhibited L74M/E138A/G140S/Q148H with an antiviral potency of 24.3 ± 3.0 nM, which was significantly better when compared to BIC (102.5 ± 13.6 nM; *p*-values < 0.001) and DTG (126.6 ± 6.2 nM; *p*-values < 0.001). In addition, 4d inhibited the IN quadruple mutant L74M/E138K/G140S/Q148H with a better antiviral potency (24.4 ± 3.4 nM) than BIC (52.9 ± 8.2 nM; *p*-values < 0.01) and DTG (163.4 ± 11.0 nM; *p*-values < 0.001). Compound 4d (26.8 ± 3.8 nM) was more effective against the IN quadruple mutant L74I/E138A/G140S/Q148H. 4d (26.8 ± 3.8 nM) was also more potent when compared to BIC (59.3 ± 6.7 nM; *p*-values < 0.001) and DTG (78.5 ± 7.3 nM; *p*-values < 0.001). Finally, 4d was also more potent (26.0 ± 3.7 nM) at inhibiting the quadruple mutant L74I/E138K/G140S/Q148H when compared to BIC (48.7 ± 4.5 nM; *p*-values < 0.001) and DTG (81.6 ± 3.6 nM; *p*-values < 0.001).

### 3.2. Replication of IN Quadruple Mutants in a Single-Round Infection Assay

Single-round assays measure the ability of the HIV-1 (WT or mutant) to infect cells only once as opposed to multi-round replication competent HIV-1. Although multi-round infection assays are quite sensitive to the effects of a mutation (or mutations) on the ability of the virus to replicate, it is difficult to control for effects of the mutation(s) on the length of the viral life cycle, which can affect the number of rounds of infection, which, in turn, affects the results. In general, all IN mutants have a reduced ability to infect when compared to WT HIV-1 in single-round infection assays [15]. To determine the magnitude of the effects of the mutations on HIV-1 infection, we measured the ability of the HIV-1 IN quadruple mutants to infect HOS cells in a single-round infection assay (Table 2). All eight IN quadruple mutants had reduced abilities to infect cells in the assay. However, with one exception, the infectivity of the quadruple mutants was >50% of WT in our single-round assay. There were no statistically significant differences when we compared the abilities of the different mutants to infect. We generated and tested a related set of quintuple mutants; however, none of the mutants we tested showed detectable infectivity. Thus, these new IN quadruple mutants can infect reasonably well, albeit with a reduction of ~30–40%, when compared to WT HIV-1 in a single round.

## 4. Discussion

Second-generation INSTIs are typically recommended to treat both treatment-naïve and treatment-experienced PLWH, and the development of resistance to this class of INSTIs has been infrequent in treatment-naïve PLWH. However, the emergence of resistance, specifically in treatment-experienced, INSTI-experienced PLWH has been reported, and there have been additional reports of DTG-resistance mutations [12,13,25]. With wider use of the second-generation INSTIs in low- and middle-countries, there has been an increase in transmission of HIV-1 drug resistance, and we should expect to see an increase in the number and diversity of IN mutants.

In the current work, we evaluated the antiviral potencies of second-generation INSTIs and compound 4d against a panel of eight IN quadruple mutants. These IN mutants have multiple mutations at key positions around the IN active site. Recent work has shed light on mechanisms of resistance when mutations occur at IN positions E138K, G140A/S, and Q148H/K/R in both SIVrcm IN and HIV-1 IN [26,27]. In this report, we added either L74I/M, E92Q, or T97A to E138A/K/G140S/Q148H to generate quadruple mutants. These particular IN mutants have been identified in clinical trials where PLWH have been put on a DTG-containing regimen after failing a first-generation raltegravir-based therapy [12,13]. Overall, 4d showed improved potencies against five out of the eight IN mutants in this panel when compared to the second-generation INSTIs. The only time that BIC displayed greater potency compared to 4d was against the IN mutant E92Q/E138K/G140S/Q148H. However, the fact that none of the INSTIs retained high potencies against the IN mutants we tested is troubling.

Moving forward, it will be important to understand how acquiring a fourth mutation affects both the E138A/G140S/Q148H and E138K/G140S/Q148H mutants. The addition of the fourth mutation causes a 5-fold decrease in potency against five of the eight IN quadruple mutants and a 10-fold decrease against the remaining IN quadruple mutants for BIC when compared to antiviral potencies against E138A/G140S/Q148H or E138K/G140S/Q148H. DTG is largely ineffective when the fourth mutation is added, and it exhibits between 15-fold and 30-fold loss in potency when compared to its potencies against the IN triple mutants. Furthermore, CAB is compromised and completely ineffective when a fourth mutations is added to E138A/G140S/Q148H or E138K/G140S/Q148H. Compound 4d only showed a modest loss of potency of ~5-fold against five of the eight IN quadruple mutants, but it is largely ineffective when E92 is the fourth mutation [16,21]. These antiviral data highlight the importance of understanding how the fourth mutation affects the mutant intasome inhibitor interface. At the same time, these large losses in potencies are quite alarming and pose a potential problem for HIV-1 patients.

The ways in which the IN mutations affect the binding of the INSTI to the active site of the HIV intasome are not well-understood. It is likely that mutations at IN positions L74, E92, or T97 disrupt interactions that feed back to IN catalytic residue D64, as has been previously reported [28]. The IN residue L74, which is in the catalytic core domain (CCD) β-strand 2, interacts with a variety of residues including, but not limited to, L63, C65, A86, E87, T97, F100, and F121 (Figure S1). The IN residue T97 within the CCD α-helix 1 interacts with A86, E96, A86, L101, and F121 (Figure S1), while E92 within the IN CCD β3α1 loop interacts with H67 within the CCD β-strand 1 as well as with V72 and N120 (Figure S2). As previously noted, most of these interactions are located within a hydrophobic cluster near the IN active site. A mutation at any one of these positions could destabilize the antiparallel beta sheet (β-strands 1, 2, and 3) in the CCD. This could potentially affect the positioning of the IN β-strand 1 catalytic residue D64 and alter the orientation of its bi-coordination of the two Mg^2+^ ions in the active site of the HIV-1 intasome. This, in turn, could affect INSTI chelation. We hypothesize that several structural attributes of 4d affect its ability to bind in the active site of the HIV-1 intasome. These include its naphthyridine scaffold, which results in closer proximity in binding to Mg^2+^ ions, and increased π-π stacking with the terminal adenine on the viral DNA end.

The ability of 4d to retain considerable antiviral potency against a panel of IN quadruple mutants is encouraging. However, the panel has identified IN mutants that display large reductions in susceptibility to 4d. A primary focus of our current work is to identify INSTIs that retain strong antiviral potency against the IN double mutant G140S/Q148H. We believe that this IN double mutant is a major gateway to increased INSTI resistance. The availability of structural data of INSTIs bound to intasomes containing these mutations should greatly facilitate developing new INSTIs with improved antiviral profiles.

## Figures and Tables

**Table 1:** Antiviral potencies of compound 4d and the second-generation INSTIs against a panel of IN quadruple mutants with mutations at IN positions L74, E92, or T97 combined with E138A/K/G140S/Q148H. The antiviral potencies of the second-generation INSTIs and 4d against IN the triple mutants E138A/G140S/Q148H and E138K/G140S/Q148H were determined previously [16,21] and are included with current antiviral data against the eight IN quadruple mutants to facilitate comparison. The table shows the numerical values of the EC_50_ values (in nM) and the standard deviations (n = 3) of the FDA-approved second-generation INSTIs and 4d against the panel of IN quadruple mutants.

WT or IN Mutant	BIC	DTG	CAB	4d
WT	2.4 ± 0.4 nM	2.6 ± 0.3 nM	1.8 ± 0.5 nM	1.5 ± 0.3 nM
E138A/G140S/Q148H	5.1 ± 0.5 nM	13.8 ± 4.8 nM	70.2 ± 9.0 nM	7.3 ± 0.4 nM
E138K/G140S/Q148H	4.7 ± 1.0 nM	10.3 ± 0.6 nM	44.7 ± 1.6 nM	4.5 ± 0.3 nM
E92Q/E138A/G140S/Q148H	102.5 ± 13.6 nM	280.1 ± 29.4 nM	952.1 ± 15.7 nM	120.9 ± 22.6 nM
E92Q/E138K/G140S/Q148H	78.5 ± 10.9 nM	126.5 ± 31.0 nM	513.8 ± 77.9 nM	179.2 ± 27.6 nM
T97A/E138A/140S/Q148H	126.0 ± 6.0 nM	175.5 ± 19.8 nM	2903 ± 313.3 nM	92.5 ± 4.7 nM
T97A/E138K/140S/Q148H	64.3 ± 4.5 nM	221.8 ± 25.9 nM	1989.3 ± 306.3 nM	52.1 ± 8.8 nM
L74M/E138A/G140S/Q148H	102.5 ± 13.6 nM	126.6 ± 6.2 nM	656.2 ± 54.0 nM	24.3 ± 3.0 nM
L74M/E138K/G140S/Q148H	52.9 ± 8.2 nM	163.4 ± 11.0 nM	541.3 ± 75.9 nM	24.4 ± 3.4 nM
L74I/E138A/G140S/Q148H	59.3 ± 6.7 nM	78.5 ± 7.3 nM	677.9 ± 26.5 nM	26.8 ± 3.8 nM
L74I/E138K/G140S/Q148H	48.7 ± 4.5 nM	81.6 ± 3.6 nM	699.1 ± 89.3 nM	26.0 ± 3.7 nM

**Table 2:** Infection of IN quadruple mutants in a single-round infection assay. The relative levels of infection by the IN mutants in a single-round infection assay are shown with standard deviations, n = 3.

WT or IN Mutant	Single-Round Infectivity (% of WT)
WT	100
E92Q/E138A/G140S/Q148H	57.0 ± 12.2
E92Q/E138K/G140S/Q148H	63.0 ± 8.5
T97A/E138A/G140S/Q148H	56.7 ± 5.8
T97A/E138K/G140S/Q148H	61.0 ± 7.3
L74M/E138A/G140S/Q148H	57.0 ± 12.3
L74M/E138K/G140S/Q148H	67.0 ± 8.5
L74I/E138A/G140S/Q148H	72.0 ± 1.6
L74I/E138K/G140S/Q148H	70.0 ± 12.6

## Data Availability

Data will be made available upon request.

## Supplementary Materials

The following supporting information can be downloaded at https://www.mdpi.com/article/10.3390/v17010121/s1, Figure S1: Location of IN residues L74 and T97; Figure S2: Location of IN residue E92 in the IN active site.
