# Supplementary material for: Comparative Analyses of Antiviral Potencies of Second-Generation Integrase Strand Transfer Inhibitors (INSTIs) and the Developmental Compound 4d Against a Panel of Integrase Quadruple Mutants
Source: Viruses. 2025 Jan 16;17(1):121. doi: 10.3390/v17010121 (PMC11768864; doi:10.3390/v17010121)
Supplement: Supplementary file 1 [file viruses-17-00121-s001.zip › viruses-3382002-supplementary.pdf]

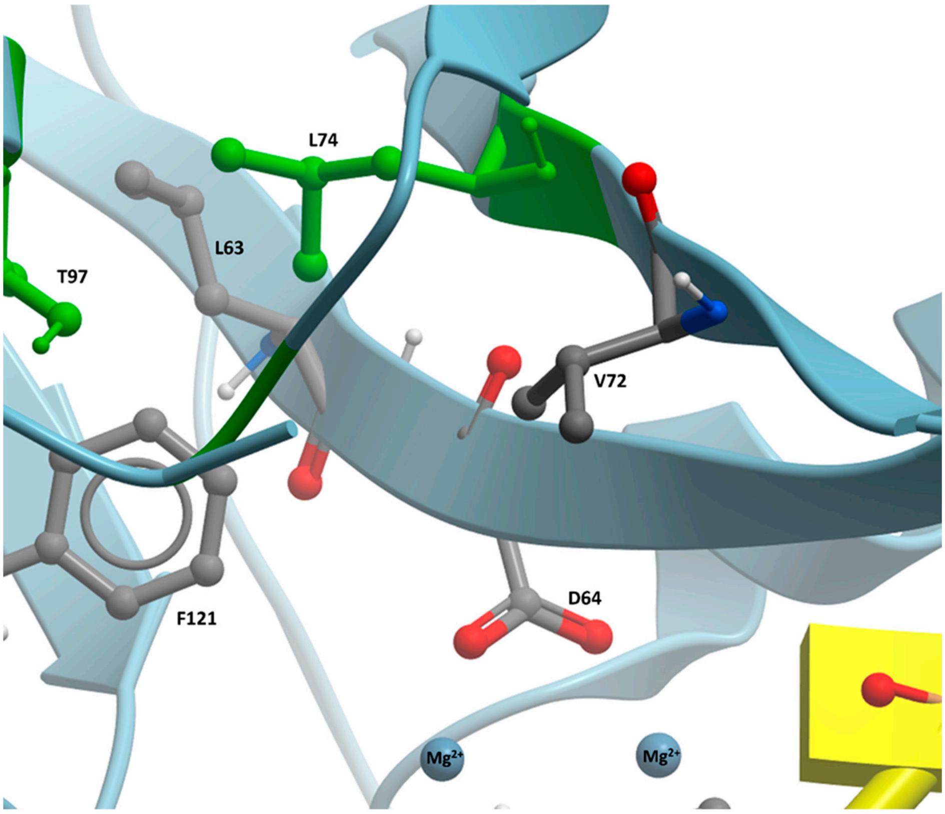

Supplementary Figure S1. Location of IN residues L74 and T97. A model of IN residues L74 and T97 are shown in green around the IN active site. Nearby residues, L63, V72, and F121, that are close in proximity to IN active site D64 are noted (in black). Mg^{2+} are also shown and labeled.

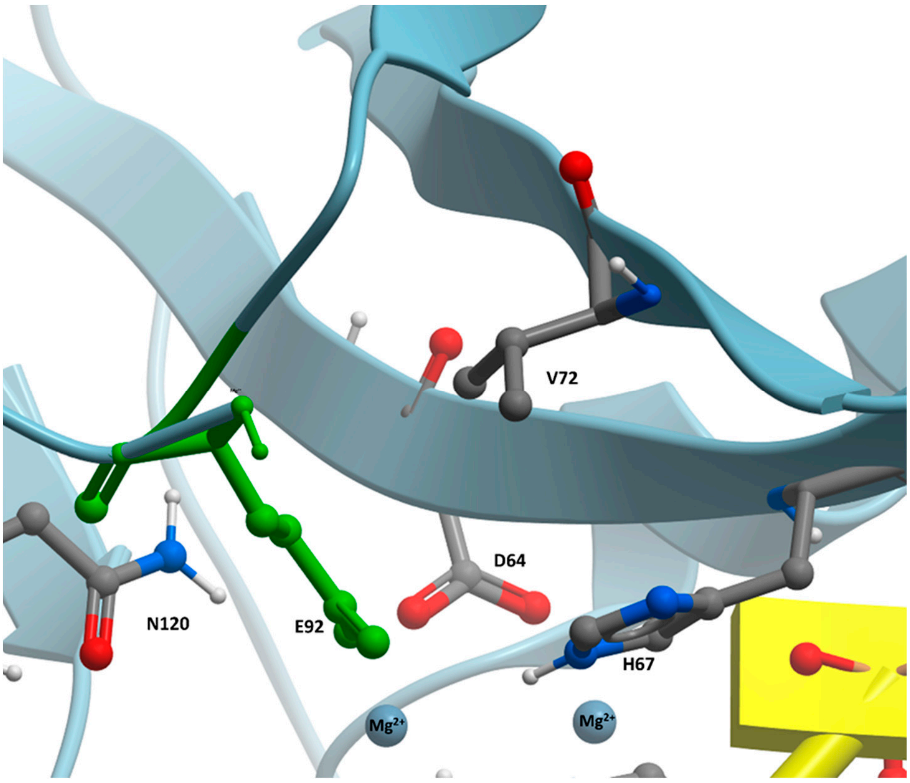

Supplementary Figure S2. Location of IN residue E92 in the IN active site. A model of IN residue E92 is shown in green around the IN active site. Nearby residues, H67, V72, and N120 that reside close to IN active site D64 are noted (in black). Mg^{2+} are also shown and labeled.
